# Noise-Related Hearing Disorder Among Vector Control Workers in Kuala Lumpur

**DOI:** 10.7759/cureus.46965

**Published:** 2023-10-13

**Authors:** Kian Kiong Yap, Nor Halizam Ismail, Rama Krishna Supramanian, Yin Cheng Lim

**Affiliations:** 1 Social Preventive Medicine, University Malaya, Kuala Lumpur, MYS; 2 Health and Environment, Kuala Lumpur City Hall, Kuala Lumpur, MYS

**Keywords:** noise-induced hearing loss, fogging activities, hearing, noise exposure, audiometry

## Abstract

Introduction

Noise-related hearing disorder (NRHD) is the second most common sensorineural hearing loss, right after age-related hearing loss (presbycusis). It is the highest reported occupational disease and a major compensable occupational hazard in Malaysia. With the increase in dengue cases, the need for vector control workers to control the spread of dengue at the expense of being exposed to noisy fogging machines is critical.

Methods

This was a cross-sectional study of vector control workers conducted by a local authority in Kuala Lumpur. Participants were categorised as either vector control workers who were directly involved in fogging activities or those who were not. A self-administered questionnaire was used to collect sociodemographic, medical and occupational information. NRHD was confirmed by an audiogram test.

Results

This study found a high prevalence of NRHD among vector control workers exposed to fogging activity (occupational noise hazards), with 51.4% of them experiencing this condition. The predictors of NRHD include fogging status adjusted odds ratio (aOR) 1.94 (95% CI: 1.19 - 3.17), sex 18.28 (95% CI: 2.33 - 143.16) and age 2.03 (95% CI: 1.27 - 3.25).

Conclusion

The findings of this study imply that vector control workers are at risk of NRHD. The predictors of NRHD are fogging status, sex and age. These findings emphasise the major impact of occupational noise hazards on NRHD and emphasise the importance of addressing this issue to preserve employees' health; especially among male and older employees with chronic noise exposure.

## Introduction

Noise-related hearing disorder (NRHD) is the highest-reported occupational disease and a major compensable occupational hazard in Malaysia. It is categorised into three groups, as quoted by the Department of Occupational, Safety and Health (DOSH): i) noise-induced hearing loss (NIHL), ii) hearing impairment (HI) and iii) permanent standard threshold shift (STS) [1]. Prevalence of NIHL varies by occupational groups in Malaysia: traffic police personnel (80%), quarry workers (57%), airport workers (33.5%), vector control workers (26.5%) and dental staff nurses (5%). This can severely affect the employee's well-being and work performance [2]. Many occupations around the world with machinery involvement are exposed to noise, such as agriculture, aviation, carpentry, construction, food and beverages, entertainment, manufacturing, mining and quarrying, textile and vector control.

Occupational noise is defined as "a disturbance of sound at a workplace received by a worker’s auditory system when they are working" [3]. According to the Centers for Disease Control and Prevention (CDC), over 22 million workers are exposed to harmful noise levels at work annually, putting not just their health and well-being in danger, but also those around them [4]. WHO reported that more than 16% of global deafness or 4 million disability-adjusted life years (DALYs) is due to work-related causes. In developing countries, the burden of NIHL among employees remains high [5]. In Malaysia, fogging using ultra-low volume insecticide particles remains a mainstream method for dengue control activities, which is part of the Ministry of Health (MOH) Integrated Vector Management (IVM) for Aedes Control adopted from WHO. Cross-sectional research was done on NIHL among vector control workers in the state of Negeri Sembilan, Malaysia. Results showed that NIHL prevalence among vector control workers was 26%. The noise level of fogging machines exceeded more than 90dB(A) at a distance of 0.5 m [6]. This demonstrated that vector control workers are a vulnerable population at risk for NRHD due to high noise levels exposure from fogging machines during fogging activities. Besides that, noise exposure also affects the non-auditory systems, which leads to various health complications, including cardiovascular health, pregnancy complications, sleep disturbance, and cognitive and emotional responses.

In addition, various research conducted worldwide mainly focuses on one of the three categories of NRHD for both association and prevalence. This is an important knowledge gap to be filled, especially in Malaysia’s occupational disease notification to DOSH based on the terms of NRHD, rather than individual categories. This allows a better understanding of the causal relationship between noise exposure and NRHD.

Furthermore, limited local and international studies considered the exposure of organophosphates (OP) as a confounding factor, where several studies have shown that this increases the risk of hearing loss; the possible mechanism includes DNA damage, inflammation and oxidative stress [7,8]. In addition, most studies only investigated the associated factors of NIHL rather than the predictors of NIHL; where the predictors can be utilised to create a targeted, hearing conservation program among noise-exposed workers to reduce the NIHL among the workforce. Mitigation techniques might be adapted to the occupation to guarantee an effective public health measure.

NRHD is preventable, thus it has a high importance in public health. A noisy work environment has been the largest compensable work-related hazard. However, there have been limited studies on NRHD in Malaysia despite the major implications on employee health, productivity and safety, and not least, the cost to employers. The objective of this study is to determine the predictors of NRHD among vector control workers.

## Materials and methods

Study design

This was a cross-sectional study conducted between November 2022 and February 2023 among local authority vector control workers in Kuala Lumpur. Participants were categorised as either vector control workers who were directly involved in fogging activities (those who were assigned and frequently participated in performing vector control duties, which required the operation of vector control fogging machines using pesticides in spraying and fogging operations, and where the noise risk assessment indicated Noise Exposure Limit, requiring annual audiometry testing and hearing protection for workers, as stipulated by Occupational Safety and Health {Noise Exposure} Regulations 2019) or those who were not. Everyone in the vector control division was invited to participate. Workers were excluded if they had less than 5 years of experience in their current job, worked in noisy part-time jobs, had prior pre-employment hearing impairment, were pregnant, had a history of head trauma requiring hospitalization, had a history of non-occupational trauma or accident resulting in permanent ear injury, had a history of ototoxic drug use and a history of infection (mumps/measles or tuberculosis). From participants who fulfilled the screening criteria, data were collected using self-administered questionnaires which included sociodemographic, medical and occupational information. NRHD was confirmed by an audiogram test on the participants.

Sample size

The sample sizes were calculated using NRHD (NIHL, HI, STS) as the main outcome with the significant level (α) preset at 0.05 and the power of the study as 80%. The proportion of NRHD among vector control workers who conducted fogging activities was set at 19.9% [6,9,10]. The design effect was set to 1.0. The total sample size was 245 participants.

Data collection

The collection was conducted at the local authority headquarters in Kuala Lumpur from 8:00 a.m. to 5:00 p.m. Self-administered questionnaires and audiograms were utilised for data collection.

Questionnaire

The questionnaire included data on sociodemographic, medical, occupational and non-occupational history, and smoking status ever or never). Personal protective equipment (PPE) compliance data were collected using an adapted questionnaire created by Badran et al. [11]. The degree of compliance assessment included 14 compliance items with a scale of 0 to 4 points (0 = never, 1 = rarely, 2 = sometimes, 3 = usually, and 4 = always). A score of > 2 indicates good compliance. Recreational noise exposure data were collected using an adapted questionnaire created by Armitage et al. [12]. These include headphones/earphones, live music, nightlife, sports-related noise or cinema. Any exposure once a month and above for the past 12 months indicates high risk while any exposure less than once a month and above for the past 12 months indicates low risk. Environmental noise exposure data was collected using an adapted questionnaire developed by Rama et al. [2]. Place of residence located in a place with loud noise (for example, near a highway, factory or airfield) indicates a positive exposure.

The questionnaire was translated from English to Malay by two bilingual (English and Malay language) public health authors and the Institute of Language and Literature, Malaysia (DBP). Subsequently, two different bilingual public health authors translated back to the Malay language into English, using the back-to-back translation method. The finalised version of the Malay questionnaire was reviewed by two public health practitioners for content validity and to ensure satisfactory face, criterion and conceptual equivalent. All vector control workers were briefed about the study, which consist of the research purpose, objectives and benefits of the research. Workers who agreed to participate were given a screening questionnaire to determine their eligibility based on the exclusion criteria of this study. All returned screening questionnaires were screened for completeness. Participants who fulfilled the criteria were allowed to proceed to the next phase. Social-demographic questionnaires are given to eligible employees at their workplace. Questionnaires were returned and collected upon completion by the agreed participants. They were screened for completeness and were kept in a manual filling system.

Audiometry

Audiometry testing was conducted by a trained occupational staff nurse to assess NRHD. Hearing threshold levels of the participants were assessed at frequencies of 500, 1000, 2000, 3000, 4000, 6000 and 8000 Hz. It is recorded for each ear at each frequency and measured in decibels (dB) with 0 decibel representing average hearing ability for adults with no ear pathology. Larger threshold values indicate poorer-than-average hearing. Audiometry was performed in an audiometric booth at the local authority staff clinic. All audiometric booths had undergone periodic calibration and were certified by DOSH. Prior to testing, participants were required to have a 14-hour silent period and those who displayed any upper respiratory tract infection (URTI) symptoms were rescheduled. Prior to audiometry, the auditory canal and tympanic membrane were examined to rule out conductive hearing loss caused by impacted earwax and perforated tympanic membrane. They are reviewed accordingly to determine whether the participants were diagnosed with NRHD (a “threshold dip”/“hearing notch” between 4000-6000 Hz OR arithmetic average of the permanent hearing threshold level of an employee at 500, 1000, 2000 and 3000 Hz which is shifted by 25 dB or more compared to the standard audiometric reference level OR change in the hearing threshold compared to the baseline audiogram of an average of 10 dB or more at 2000, 3000 and 4000 Hz on pure-tone audiometry testing), which includes NIHL, HI and STS. They were grouped into vector control workers who were directly involved in fogging activities and those who were not. Missing data are excluded.

Statistical analysis

Data was analysed using Statistical Package for the Social Science (SPSS) version 28.0 (IBM Corp., Armonk, NY). Descriptive analysis is used to describe vector control worker's sociodemographics. Chi-Square (x2) was used to determine the associated factors of NRHD. Multivariable logistic regression to determine the association between NRHD and its predictors (fogging activities {occupational noise exposure}, sex, age, race, marital status, income group, education level, smoking status, hypertension, diabetes mellitus, dyslipidaemia, recreational noise exposure, environmental noise exposure) among vector control workers. Further analysis was also conducted where fogging status is atomised according to the Duration of Noise Exposure and PPE Compliance among vector control workers who conduct fogging activities. Adjusted odds ratios (aOR) and 95% confidence interval were used to determine the strength of associations between the variables. Statistical significance was set at a p-value less than 0.05.

Ethics approval and consent to participate

Ethical approval for this study is granted and registered with the National Medical Research and Ethics Committee (NMRR ID-22-01856-37U). Informed consent was obtained from all individual participants included in the study.

## Results

Characteristics of subjects

**Figure 1:**
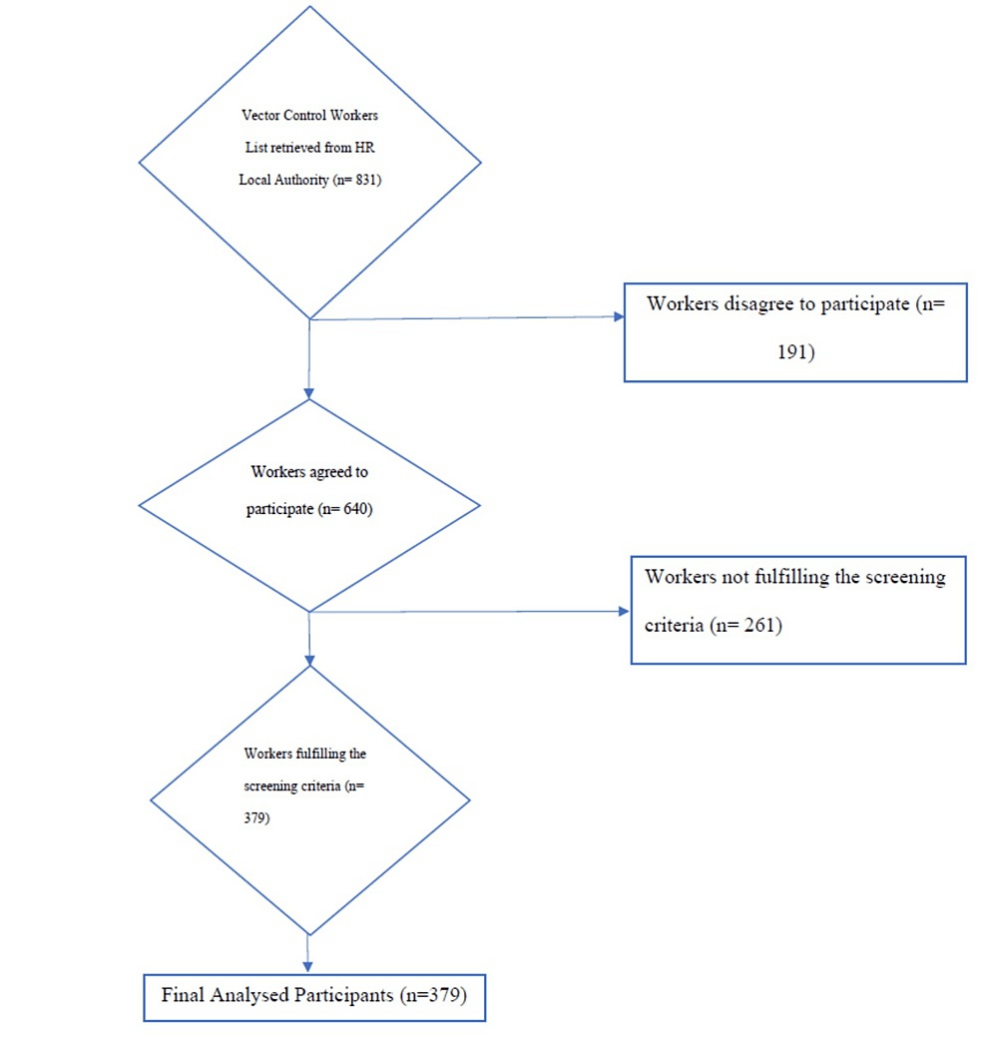
Inclusion and Exclusion of Participants

A sum of 191 workers disagreed to participate in the study out of the 831 workers. Out of the 640 participants, 261 workers failed to fulfil the screening criteria to participate in the study. The final number of 379 participants was analysed (Figure 1).

Around 62.3% of the study participants’ age are under the age of 40 years old. There are 94.5% male and 5.5% female among the participants. For lifestyle characteristics, 36.7% are non-smokers. In terms of medical history, 16.6% have hypertension, 13.2% have diabetes, and 10.6% have dyslipidaemia. Only a minority reported having been exposed to environmental noise and recreational noise exposure, standing at 9.8% and 6.5% respectively. These are shown in Table 1.

**Table 1:** Characteristics of Participants (n=379) PPE: Personal Protective Equipment *: p-value < 0.05

Variables	Overall	Fogging	No Fogging	
	(n=379)	(n=259)	(n=120)	
	n (%)	n (%)	n (%)	p-value
Sociodemographic				
Age (years)				
≤ 40	236 (62.3)	173 (66.8)	63 (52.5)	< 0.05*
> 40	143 (37.7)	86 (33.2)	57 (47.5)	
Gender				
Male	358 (94.5)	253 (97.7)	105 (87.5)	< 0.05*
Female	21 (5.5)	6 (2.3)	15 (12.5)	
Ethnicity				
Malay	321 (84.7)	231 (89.2)	90 (86.6)	< 0.05*
Chinese		0 (0.0)	0 (0.0)	
Indian	58 (15.3)	28 (10.8)	30 (12.6)	
Others		0 (0.0)	0 (0.0)	
Marital Status				
Single	39 (10.3)	24 (9.3)	15 (11.2)	0.256
Divorced	6 (1.6)	6 (2.3)	0 (0.0)	
Widower/Widow	7 (1.8)	4 (1.5)	3 (1.6)	
Married	327 (86.3)	225 (86.9)	102 (85.0)	
Education Level				
Primary	38 (10.0)	16 (6.2)	21 (17.5)	< 0.05*
Secondary	324 (85.5)	235 (90.7)	90 (75.0)	
Tertiary	17 (4.5)	8 (3.1)	9 (7.5)	
Income Group (Ringgit Malaysia-RM)				
B1 (< 2500)	188 (49.6)	133 (51.4)	55 (45.8)	< 0.05*
B2 (2500-3169)	107 (28.2)	77 (29.7)	30 (25.0)	
B3 (3170-3969)	55 (14.5)	34 (13.1)	21 (17.5)	
B4 (3970-4849)	25 (6.6)	15 (5.8)	10 (8.3)	
M1 (4850-5879)	4 (1.1)	0 (0.0)	4 (3.3)	
Hypertension				
Yes	63 (16.6)	35 (13.5)	28 (23.3)	< 0.05*
No	316 (83.4)	224 (86.5)	92 (76.7)	
Diabetes				
Yes	50 (13.2)	28 (10.8)	22 (18.3)	< 0.05*
No	329 (86.8)	231 (89.2)	98 (81.7)	
Dyslipidaemia				
Yes	40 (10.6)	25 (9.7)	15 (12.5)	0.401
No	339 (89.4)	234 (90.3)	105 (87.5)	
Smoking				
Daily	126 (33.2)	90 (34.7)	36 (30.0)	< 0.05*
Occasional	63 (16.6)	55 (21.2)	8 (6.7)	
Former	51 (13.5)	35 (13.5)	16 (13.3)	
Never	139 (36.7)	79 (30.5)	60 (50.0)	
Occupational Characteristic				
Job Scope				
Fogging Activities		259 (100)	-	-
Administrative		-	4 (3.0)	
Driver		-	2 (2.0)	
General Fieldworker		-	48 (40.0)	
Larvicider		-	36 (30.0)	
Maintenance Crew		-	12 (10.0)	
Supervisor		-	12 (10.0)	
Duration of Noise Exposure				
≤ 10 years		118 (31.1)	-	-
> 10 years		141 (37.2)	-	
PPE Compliance				
Yes		248 (95.8)	-	-
No		11 (4.2)	-	
Organophosphate Poisoning				
Yes		0	-	-
No		259 (100.0)	-	
Types of Fogging Machines Used				
i) Agrofog				
Yes		254 (67.0)	-	-
No		5 (1.3)	-	
ii) Swingfog				
Yes		46 (12.1)	-	-
No		213 (56.2)	-	
Non-occupational Noise Exposure				
Environmental				
Yes	37 (9.8)	24 (9.3)	13 (10.8)	0.633
No	342 (90.2)	235 (90.7)	107 (89.2)	
Recreational				
High	18 (4.7)	10 (3.9)	8 (6.7)	< 0.05*
Low	7 (1.8)	2 (0.8)	5 (4.2)	
No	354 (93.4)	247 (95.4)	107 (89.2)	

Of the total participants, 259 (68.3%) were vector control workers directly involved in fogging activities. Among those who were exposed to fogging activities (noisy work environment), 31.1% of workers had been exposed 10 years and below while 37.2% had been exposed more than 10 years. 37.2% had been employed for a duration of more than 10 years while 95.8% had used their PPE. None of the vector control workers had organophosphate poisoning. Sixty-seven percent of the vector control workers who were directly involved in fogging activities used Argofog while 12.1% used Swingfog (both, commonly used fogging machine brands in Malaysia).

Effect of noise exposure on the risk of NRHD and its predictors

Table 2 shows the relationship between various factors and NRHD, analysed using univariate analysis. The result showed that vector control workers who were directly involved in fogging activities are at a higher risk of developing NRHD with an OR of 1.76 (95% CI: 1.13 - 2.74) compared to those who were not, with a p-value of 0.012.

**Table 2:** Univariate Analysis of Noise-Related Hearing Disorder (NRHD) and its Confounding Factor NRHD: Noise-Related Hearing Disorder; OR: Odds Ratio; CI: Confidence Interval

Variables	NRHD	OR	95% CI	p-value	
Sex					
Female	1 (0.6)	1.00 (Ref)			
Male	177 (99.4)	19.56	2.60 - 147.29	< 0.001	
Age					
≤ 40	94 (52.8)	1.00 (Ref)			
> 40	84 (47.2)	2.15	1.41 - 3.28	< 0.001	
Race					
Non-Malay	33 (18.5)	1.00 (Ref)			
Malay	145 (81.5)	0.62	0.36 - 1.10	0.100	
Marital Status					
Married	154 (86.5)	1.00 (Ref)			
Non-married	24 (13.5)	0.96	0.54 - 1.73	0.900	
Income Group (Ringgit Malaysia-RM)					
M40/T20 (> 4849)	2 (1.1)	1.00 (Ref)			
B40 (≤ 4849)	176 (98.9)	0.88	0.12 - 6.35	0.903	
Education Level					
> Primary school	154 (86.5)	1.00 (Ref)			
Primary school	24 (13.5)	2.08	1.04 - 4.16	0.035	
Smoking Status					
Non-smoker	60	1.00 (Ref)			
Smoker/ Ex-smoker	118	1.27	0.84 - 1.94	0.259	
Hypertension					
No	145	1.00 (Ref)			
Yes	33	1.30	0.76 - 2.23	0.346	
Diabetes Mellitus					
No	151	1.00 (Ref)			
Yes	27	1.38	0.76 - 2.52	0.285	
Dyslipidaemia					
No	156	1.00 (Ref)			
Yes	22	1.43	0.74 - 2.77	0.282	
Fogging Status					
No	45 (25.3)	1.00 (Ref)			
Yes	133 (74.7)	1.76	1.13 - 2.74	0.012*	
Environmental Noise					
No	161 (90.4)	1.00 (Ref)			
Yes	17 (9.6)	0.96	0.48 - 1.89	0.896	
Recreational Noise					
No	170 (95.5)	1.00 (Ref)			
Yes	8 (4.5)	0.90	0.35 - 2.33	0.826	

Besides fogging activities (occupational noise) exposure, various other factors were discovered to be significantly associated with NRHD in this study. This included age, sex and education level. Workers aged 40 years or older had an odds of developing NRHD with an OR of 2.15 (95% CI: 1.41 - 3.28) compared to workers under the age of 40.

Male workers had an odds of developing NRHD with an OR of 19.56 (95% CI: 2.60 - 147.29) compared to female workers. Similarly, workers who had primary education had an odds of developing NRHD with an OR of 2.08 (95% CI: 1.04 - 4.16) compared to workers who had secondary education and above.

In contrast, various factors that were examined were not found to be significantly associated with NRHD in this study. These included race, marital status, income level, smoking status, hypertensive status, diabetic status, dyslipidaemic status, and environmental and recreational noise exposure. 

Table 3 shows the association between fogging activities (occupational noise exposure) and NRHD. From the univariate logistic regression in Table 2, five variables are considered included in the multivariable logistic regression model using p-value < 0.25: fogging status, sex, age, race, and education level. After the five variables (fogging status, sex, age, race, and education level) were included in the multivariable logistic regression model, only three variables were statistically significant (p-value < 0.05), which is fogging status, sex and age as shown in Table 3. The model has a satisfactory fit as demonstrated by the Hosmer-Lemeshow test of p > 0.514 (p > 0.05 indicates no difference between predicted and observed values) with a classification table of 64.4%.

**Table 3:** Multiple Logistic Regression of the Predictive Factors of Noise-Related Hearing Disorder (NRHD) Among Vector Control Workers Sig: Significant Value; aOR: Adjusted Odds Ratio; CI: Confidence Interval HL Test: p-value > 0.514 (> 0.05)

Predictors	Coefficient	Standard Error	p-value	aOR (95% CI)
Fogging Status				
No				1.00 (Ref)
Yes	0.66	0.25	0.008*	1.94 (1.19 - 3.17)
Sex				
Female				1.00(Ref)
Male	2.91	1.05	0.006*	18.28 (2.33 - 143.16)
Age				
≤ 40				1.00 (Ref)
> 40	0.71	0.24	0.003*	2.03 (1.27 - 3.25)
Race				
Non-Malay				1.00 (Ref)
Malay	-0.44	0.34	0.192	0.64 (0.33 - 1.25)
Education Level				
> Primary school				1.00 (Ref)
Primary school	0.52	0.40	0.189	1.68 (0.77 - 3.66)
Constant	- 3.34	1.07	0.002	0.036

Following further analysis according to Duration of Noise Exposure and PPE Compliance among vector control workers who conduct fogging activities (noise-exposed employees), after adjusting for sociodemographic status, medical conditions and non-occupational noise exposure (Recreational Noise, Environmental Noise), the risk of NRHD was higher among employees who had a longer duration of noise exposure and non-compliance to PPE, compared to those who did not and did respectively. However, the differences were statistically significant with an OR of 2.34 (95% CI: 1.39 - 4.10) for the risk of NRHD among noise-exposed employees who were exposed to a longer duration of noise. For non-compliance to PPE, it is not statistically significant with an OR of 1.85 (95% CI: 0.43 - 8.04). The model showed a good fit to the data as demonstrated by the Hosmer-Lemeshow test of p > 0.05. These are summarized in Table 4 and Table 5 respectively.

**Table 4:** Association Between Occupational Noise Exposure and NRHD According to Duration of Noise Exposure *aOR: Adjusted Odds Ratio, adjusted for Sociodemographic Status, Medical Conditions and Non-occupational Noise Exposure Hosmer-Lemeshow Test: p-value > 0.05

Variables	aOR* (95% CI)	p-value
Duration of Noise Exposure (years)		
No	1.00 (Ref)	
i) 5-10	1.11 (0.59 - 2.09)	0.760
ii) > 10	2.04 (1.26 - 3.29)	0.004

**Table 5:** Association Between Occupational Noise Exposure and NRHD According to PHP Usage Among Noise-Exposed Employees *aOR: Adjusted Odds Ratio, adjusted for Sociodemographic Status, Medical Conditions and Non-occupational Noise Exposure; PHP: Personal Hearing Protectors Hosmer-Lemeshow Test: p-value > 0.05

Variables	aOR* (95% CI)	p-value
PHP Usage		
Yes	1.00 (Ref)	
No	0.58 (0.26 - 1.27)	0.171

## Discussion

This report established that, after controlling for various confounding variables, vector control workers who were directly involved in fogging activities (occupational noise) had a considerably higher likelihood of developing NRHD compared to those who were not with an OR of 1.94 (95% CI: 1.19 - 3.17; p < 0.05). The predictors of NRHD were fogging status, sex and age. Although other variables may potentially play an essential part in NRHD development, the findings indicate that they are not significant predictors.

This finding is consistent with prior research showing that workers who are chronically exposed to continuous noise levels of 85 dB and above are more likely to have hearing disorders than those who are not exposed, supporting the hypothesis that occupational noise exposure is linked to an increased risk of NRHD. In a cross-sectional study conducted among 181 Malaysian vector control workers from Negeri Sembilan, fogging activities (occupational noise exposure) were found to be associated with NIHL (OR 6.87, 95% CI: 1.54 - 30.69) [6]. Similar results were also concluded in a study among 121 vector control workers in Sabah, positive association was demonstrated between both fogging activities (occupational noise exposure) and hearing disorder, which is in line with the conclusions made in this report [13].

There is a clear association between fogging activities and NRHD as proven in previous research and in the univariate analysis above. This is true as the noise level of fogging machines exceeded more than 90dB(A) at a distance of 0.5m. This is further proven in the annual noise risk assessment conducted by the local authority showing that for these vector control workers who conduct fogging activities, the daily personal noise dose (Dose8H) exceeds the daily excessive noise limit of 50% and the permitted daily noise dose exposure level of 100%. The same goes for age as it is a well-known risk factor for hearing loss suggested in a number of literature. Changes in the inner ear that occur as people age for physiological reasons may hasten the onset of NRHD [14]. Sex was established as a predictor in this study as the majority of the vector control workers (blue-collar workers) were male. Men have traditionally been employed in male-dominated, high-noise, manual activities and sectors [15].

PPE compliance (self-reported) showed no association with reducing the risk of NRHD among noise-exposed workers, which was in line with a prior study that demonstrated that PPE compliance (self-reported) did not lower the risk of NIHL among noise-exposed workers [13]. This could indicate that the effectiveness of PPE for noise protection is limited; although the current PPE provided to participants was sufficient in achieving an effective noise level of below 80dB(A). Knowledge is generally related to better self-care. Blue-collar workers have poor knowledge due to lower education levels, thus having less knowledge and understanding about the danger of noise hazards and the significance of PPE. This may result in poor health practices and unsafe behaviours, resulting in poorer compliance with its use [16]. On top of that, participants were observed wearing the mandatory PPE only in the presence of the supervisor, potentially biasing the findings and implying a fear of being penalised for reporting non-compliance.

This study has a few limitations. It does not include the relationship between benzidine exposure and NRHD among vector control workers. However, there is no reason to believe that the workers have any risk of benzidine poisoning as their annual medical surveillance showed no abnormal results. Future studies are recommended to understand this relationship further. Another bias that occurred in this study is known as the health worker effect. Unhealthy workers who have acquired chronic diseases or other medical illnesses may be reassigned to a different job without noise exposure or forced to retire early while healthy workers are more likely to stay in noisy work environments or live to a later age [17,18]. Because of these constraints, the true harmful effects of occupational noise on the working population may be larger than what was reported in this study. To lessen the influence of this bias, multivariate analysis was performed to compensate for potential confounders such as age. Lastly, body mass index (BMI) was not taken into consideration in this study. This factor might potentially affect the result of the present study. Diabetes is closely associated with hearing loss; however, BMI was not included in further models as it was highly correlated with diabetes due to insulin resistance, thus a direct diagnosis of diabetic status is a more acceptable measurement for determining the association of hearing loss in a given population [19]. The main strength of this study is that it is among the first few research to date that covers and studies all the diseases that fall under the NRHD spectrum rather than as an individual component. Thus, increasing accuracy and better reflecting the true association between noise exposure and NRHD within the workforce. This study also took into consideration organophosphate exposure among vector control workers, which was not conducted in other studies to date. Cofounders like hypertension, dyslipidaemia, diabetes, and environmental and recreational noise exposure were taken into consideration in this study.

## Conclusions

In general, the findings of this and previous studies demonstrated evidence beyond reasonable doubt on the positive association between fogging activities (occupational noise exposure) and the risk of NRHD, with fogging status, age and sex as it’s predictors. This must be dealt with seriously as there is an annual increase in hearing loss among vector control employees. Thus, action and prevention must be taken to address the effects of fogging machines on vector control workers to ensure an effective, efficient and healthy workforce both locally and globally.
